# Supplementary material for: Evidence supported by Mendelian randomization: impact on inflammatory factors in knee osteoarthritis
Source: Front Med (Lausanne). 2024 May 28;11:1382836. doi: 10.3389/fmed.2024.1382836 (PMC11165061; doi:10.3389/fmed.2024.1382836)
Supplement: Supplementary file 1 [file Data_Sheet_1.ZIP › data/GCST90274783_5e6/783.leaveoneout.pdf]

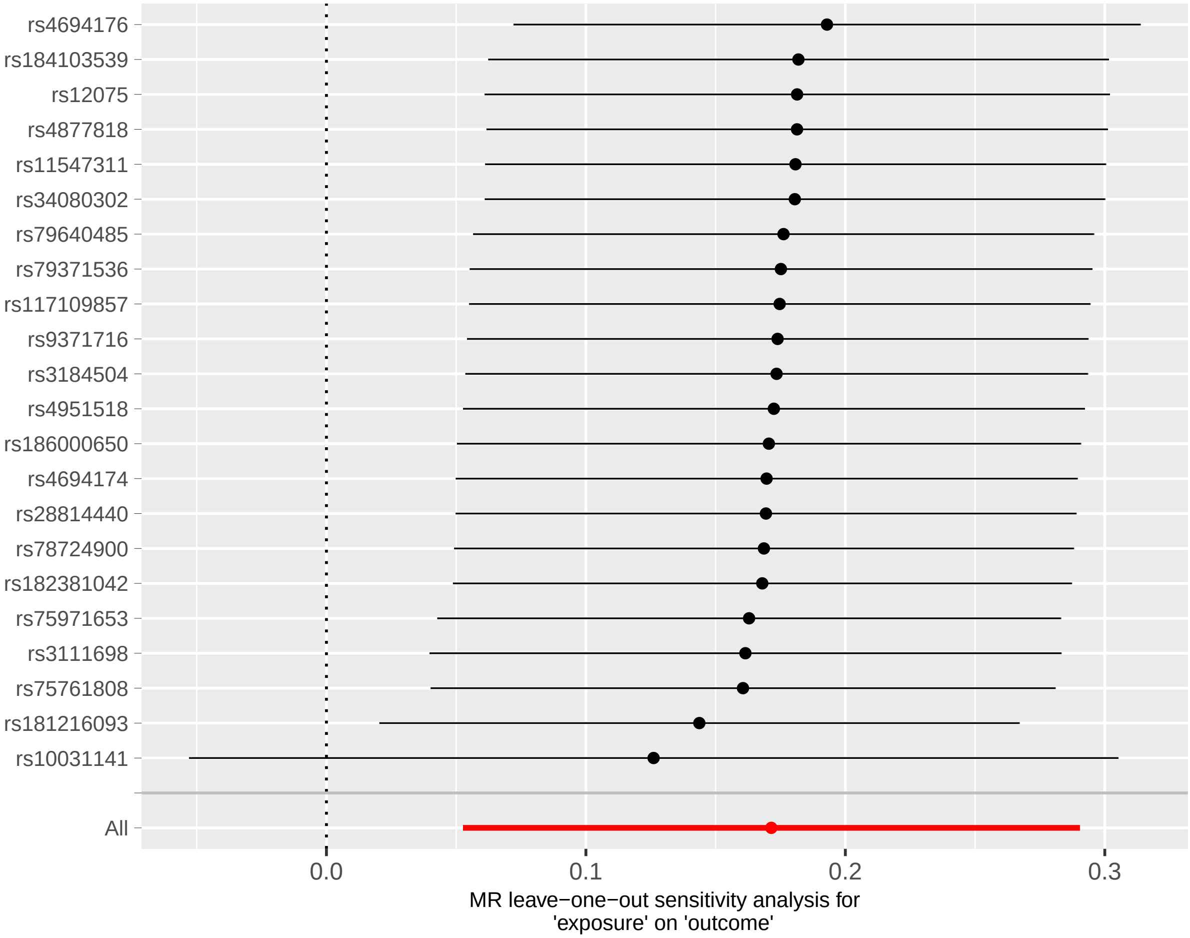

rs4694176
rs184103539
rs12075
rs4877818
rs11547311
rs34080302
rs79640485
rs79371536
rs117109857
rs9371716
rs3184504
rs4951518
rs186000650
rs4694174
rs28814440
rs78724900
rs182381042
rs75971653
rs3111698
rs75761808
rs181216093
rs10031141
All
0.0
0.1
0.2
0.3
MR leave−one−out sensitivity analysis for
'exposure' on 'outcome'
